# Bleeding duodenal ulcer after Roux-en-Y gastric bypass surgery: the value of laparoscopic gastroduodenoscopy

**DOI:** 10.4103/0256-4947.59382

**Published:** 2010

**Authors:** Hussain Issa, Osama Al-Saif, Sami Al-Momen, Bahaa Bseiso, Ahmed Al-Salem

**Affiliations:** a From the Department of Internal Medicine, King Fahad Specialist Hospital, Dammam, Saudi Arabia; b From the Department of Surgery, King Fahad Specialist Hospital, Dammam, Saudi Arabia

## Abstract

Roux-en-Y gastric bypass is a common surgical procedure used to treat patients with morbid obesity. One of the rare, but potentially fatal complications of gastric bypass is upper gastrointestinal bleeding, which can pose diagnostic and therapeutic dilemmas. This report describes a 39-year-old male with morbid obesity who underwent a Roux-en-Y gastric bypass. Three months postoperatively, he sustained repeated and severe upper attacks of upper gastrointestinal bleeding. He received multiple blood transfusions, and had repeated upper and lower endoscopies with no diagnostic yield. Finally, he underwent laparoscopic endoscopy which revealed a bleeding duodenal ulcer. About 5 ml of saline with adrenaline was injected, followed by electrocoagulation to seal the overlying cleft and blood vessel. He was also treated with a course of a proton pump inhibitor and given treatment for *H pylori* eradication with no further attacks of bleeding. Taking in consideration the difficulties in accessing the bypassed stomach endoscopically, laparoscopic endoscopy is a feasible and valuable diagnostic and therapeutic procedure in patients who had gastric bypass.

Postoperative upper gastrointestinal bleeding, though rare, is a potentially fatal complication following gastric bypass surgery.^1^–^3^ One reason for this is that endoscopic access to the excluded stomach and duodenum is difficult. A variety of techniques and diagnostic methods have been suggested to visualize the excluded stomach and duodenum. These include endoscopy via percutaneous gastrostomy, retrograde endoscopy, virtual gastroscopy using CT scan, and intraoperative gastroscopy^1^–^8^ We present a case of a recurrent bleeding peptic ulcer following gastric bypass that was diagnosed and treated with the help of laparoscopic endoscopy.

## CASE

A 39-year-old male with morbid obesity (weight=207 kg) with no other medical problems, was referred to our hospital. He underwent a Roux-en-Y gastric bypass in November 2006. Three months postoperatively, he was admitted to another hospital with melena and a drop in his hemoglobin (Hb) from 13 g/dL to 7 g/dL. He received 5 units of packed red blood cells and was started on proton pump inhibitors. He was admitted to our hospital on April 26, 2007 with similar complaints. His hemoglobin was 8.7 g/dL MCV-89.8, MCH-30.1, PLATS-258, WBC-6.1, RDW-14.3, IRON-2 umol/l. He denied any history of use of non-steroidal anti-inflammatory drugs (NSAIDs). He was given 2 units of packed red blood cells. He underwent upper gastrointestinal endoscopy as well as colonoscopy. Upper endoscopy revealed a normal esophagus; normal remnant of the stomach, with no anastomotic ulcer; and normal jejunal loop. The colonoscopy was also normal. Biopsy from the stomach remnant showed mild chronic gastritis and was positive for *Helicobacter pylori.* He was given a course of proton pump inhibitor (Esomeprazole 20 mg twice daily for I week followed by Esomeprazol 40 mg once daily for 6 weeks) and treatment for H pylori (Amoxicillin 1000 mg twice daily and Clarithromycin 500 mg twice daily for one week). There was no further bleeding and he was discharged with a hemoglobin of 9.7 g/dl. On July 22, 2007, he was readmitted with melena. Upper gastrointestinal endoscopy did not reveal the source of the bleeding. His hemoglobin on discharge was 10.4 g/dl. On February 9, 2008 he presented to our hospital with a 3-day history of melena and his hemoglobin dropped from 10.6 g/dL to 8 g/dL. He underwent an upper gastrointestinal endoscopy, which was normal. We decided to perform laparoscopic gastroduodenoscopy to evaluate the residual stomach and duodenum.

**Figure 1:**
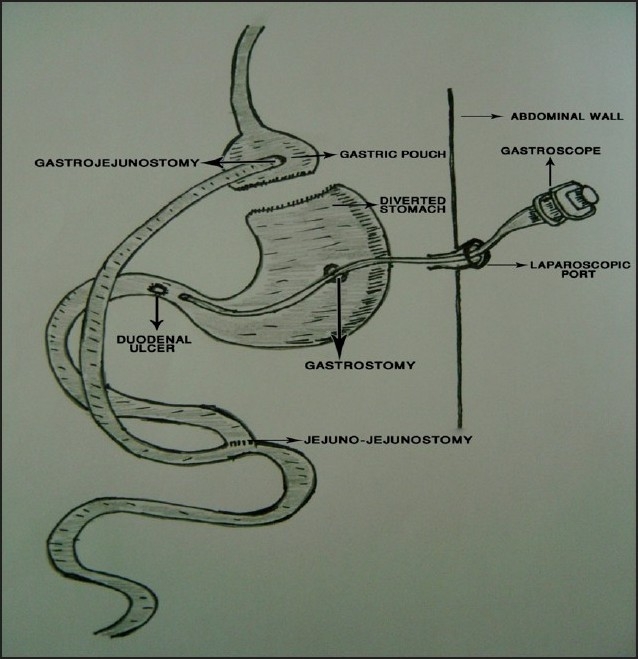
Diagrammatic representation of the laparoscopic endoscopy in our patient who had had Roux-en-Y gastric bypass for morbid obesity. The endoscope was introduced into the abdomen through a laparoscopic port and then into the diverted stomach through a gastrostomy incision.

**Figure 2:**
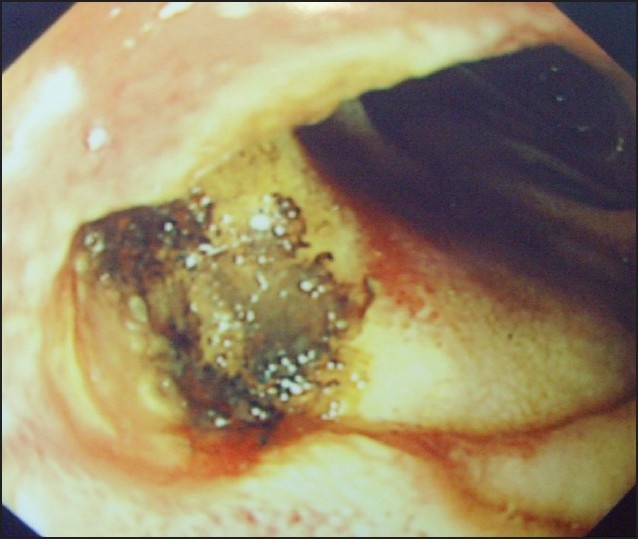
Intraoperative picture via the gastroscope showing the duodenal ulcer.

With the patient under general anesthesia, three ports were used: a size 12-mm trocar was introduced in the upper midline, below the xiphisternum, for the camera; a size 12-mm trocar was introduced in the right upper quadrant; and a size 15-mm trocar was introduced in the left upper quadrant. The residual stomach was identified and a gastrostomy was made using diathermy scissors. The gastroscope was introduced through the 15-mm trocar in the left upper quadrant and guided into the residual stomach via the already made gastrostomy (Figure 1). This revealed old blood covering the gastric mucosa. There was also a fresh duodenal ulcer with a clot over the base (Figure 2). About 5 mL of saline with adrenaline was injected, followed by electrocoagulation to seal the overlying cleft and blood vessel. The gastrostomy was closed with endo-GIA. The trocar wounds were also closed. Postoperatively, the patient did well and was started on a course of a proton pump inhibitor and given treatment for *H pylori* eradication. Ten months postoperatively he was doing well with no complaints. His follow-up hemoglobin level was 13.3 g/dL in August 2008.

## DISCUSSION

Morbid obesity is increasing worldwide. In the US, in 2002, 5.1% of adults were considered to be morbidly obese, having a body mass index of 40 or higher.^9^ This resulted in an increase in the number of gastric bypass surgeries. In the US, 13 365 bariatric surgical procedures were performed in 1998, and this increased to 205 000 in 2007.^1^,^10^ Gastric bypass is considered the most common bariatric surgical procedure, accounting for about 88% of all surgeries performed for obesity.^10^ The increase in the number of bariatric gastric bypass procedures resulted in an increase in the number of postsurgical complications. One serious and challenging complication following Roux-en-Y gastric bypass is upper gastrointestinal bleeding. It is commonly the result of bleeding from marginal ulcers at the gastrojejunal anastomosis.^11^,^12^ This has been reported in as many as 7% of all patients who have had Roux-en-Y gastric bypass.^11^ Jamil et al, in a large series of 933 patients who underwent laparoscopic Roux-en-Y gastric bypass, reported a 3.2% incidence of postoperative upper gastrointestinal bleeding.^12^ All these patients were found to have bleeding from the gastrojejunostomy staple line. The diagnosis of this complication is not difficult with the aid of esophagogastroscopy. On the other hand, a peptic ulcer in the bypassed stomach in patients who have had a Roux-en-Y gastric bypass poses both diagnostic and therapeutic difficulties. It has been shown that the bypassed gastric segment retains its ability to secrete acid and respond to vagal and hormonal stimuli.^13^,^14^ The presence of other factors such as smoking, the use of NSAIDs, and *H pylori* infection can contribute to the development of a peptic ulcer. Our patient had *H pylori* isolated from the gastric pouch. This could have contributed to the development of the peptic ulcer in the excluded stomach. These ulcers are liable to develop complications such as bleeding and perforation.^1^,^3^

The diagnosis of bleeding duodenal and antral ulcers in patients who have had Roux-en-Y gastric bypass for morbid obesity is difficult. The main reason for this is the inaccessibility of the excluded stomach because of the altered anatomy. To obviate this, lifelong proton pump inhibitors have been advocated for all patients who undergo Roux-en-Y gastric bypass.^15^ The standard gastroscope is not long enough to reach the duodenum and stomach and therefore the use of longer endoscopes has also been suggested. Upper esophagogastroscopy is however valuable to exclude marginal ulcers at the jejunogastric anastomosis. This is also the case when using transcolonic endoscopes as they are not long enough to reach the Roux limbs. To overcome this, a variety of diagnostic and therapeutic procedures have been advocated.^1^–^8^ For example a Tc-99m RBC scan or a celiac angiogram can be used to localize the site of the upper gastrointestinal bleeding; the latter can also be used to stop the bleeding once identified. Other methods that have been used include CT scan, CT angiogram, endoscopy via percutaneous gastrostomy, and intraoperative endoscopy. The technique we have described is simple and is less invasive than a laparotomy. It proved valuable as a diagnostic and therapeutic procedure.

In conclusion, upper gastrointestinal bleeding in patients who have had Roux-en-Y gastric bypass for morbid obesity presents unique diagnostic and therapeutic challenges, both for the bariatric surgeons and gastroenterologists, because of difficulties in accessing the bypassed stomach endoscopically. Laparoscopic endoscopy is a feasible and valuable diagnostic and therapeutic procedure in these patients.
